# A Metabolism-Based Interpretable Machine Learning Prediction Model for Diabetic Retinopathy Risk: A Cross-Sectional Study in Chinese Patients with Type 2 Diabetes

**DOI:** 10.1155/2023/3990035

**Published:** 2023-05-16

**Authors:** Guo-Wei Zong, Wan-Ying Wang, Jun Zheng, Wei Zhang, Wei-Ming Luo, Zhong-Ze Fang, Qiang Zhang

**Affiliations:** ^1^Department of Mathematics, School of Public Health, Tianjin Medical University, Tianjin, China; ^2^Tianjin Key Laboratory of Environment, Nutrition and Public Health, Tianjin, China; ^3^Department of Toxicology and Sanitary Chemistry, School of Public Health, Tianjin Medical University, Tianjin, China; ^4^Department of Geriatrics, Tianjin Medical University General Hospital, Tianjin Geriatrics Institute, Tianjin, China

## Abstract

The burden of diabetic retinopathy (DR) is increasing, and the sensitive biomarkers of the disease were not enough. Studies have found that the metabolic profile, such as amino acid (AA) and acylcarnitine (AcylCN), in the early stages of DR patients might have changed, indicating the potential of metabolites to become new biomarkers. We are amid to construct a metabolite-based prediction model for DR risk. This study was conducted on type 2 diabetes (T2D) patients with or without DR. Logistic regression and extreme gradient boosting (XGBoost) prediction models were constructed using the traditional clinical features and the screening features, respectively. Assessing the predictive power of the models in terms of both discrimination and calibration, the optimal model was interpreted using the Shapley Additive exPlanations (SHAP) to quantify the effect of features on prediction. Finally, the XGBoost model incorporating AA and AcylCN variables had the best comprehensive evaluation (ROCAUC = 0.82, PRAUC = 0.44, Brier score = 0.09). C18 : 1OH lower than 0.04 *μ*mol/L, C18 : 1 lower than 0.70 *μ*mol/L, threonine higher than 27.0 *μ*mol/L, and tyrosine lower than 36.0 *μ*mol/L were associated with an increased risk of developing DR. Phenylalanine higher than 52.0 *μ*mol/L was associated with a decreased risk of developing DR. In conclusion, our study mainly used AAs and AcylCNs to construct an interpretable XGBoost model to predict the risk of developing DR in T2D patients which is beneficial in identifying high-risk groups and preventing or delaying the onset of DR. In addition, our study proposed possible risk cut-off values for DR of C18 : 1OH, C18 : 1, threonine, tyrosine, and phenylalanine.

## 1. Introduction

Diabetic retinopathy (DR) is a common and specific microvascular complication of diabetes and remains the leading cause of blindness in working-aged people [1]. In a meta-analysis of 41 studies done in Chinese people between 1990 and 2017, researchers estimated that the pooled prevalence rates were 18.45% for any DR in people with diabetes which revealed that DR has been a heavy public health problem in China [2]. DR is generally asymptomatic in the early course and irreversible in the late stages. However, the therapy of DR, such as antivascular endothelial growth factor (anti-VEGF) therapy, is usually only effective in the late stages, and not all patients respond optimally [3]. There is a pressing need for new screening and treatments to prevent DR and DR-associated blindness.

Metabolites are the biological products of genomic and proteomic perturbations and also can be influenced by environment such as diet and toxins [4]. Previous metabolomic studies revealed that branched-chain amino acid (BCAA) and fatty acid (FA) might be useful to monitor the development of insulin resistance [5]. For proliferative diabetic retinopathy (PDR) in diabetic patients, the AA and acylcarnitine (AcylCN) profiles were different from non-PDR patients [4, 6, 7]. In our previous studies, tyrosine, phenylalanine, and several long-chain AcylCN were also found to be correlated with DR [8, 9]. Thus, AA and AcylCN may have potential as new biomarkers for predicting DR.

Machine learning (ML) algorithms have great generalizability and discrimination in high-dimensional data to analyze complex real-world data [10]. Some studies of predicting diabetes and its complication risk based on ML algorithms have performed great predictive results [11, 12]. However, evaluation of those studies was not enough. Moreover, due to the black box problem of ML models, we cannot know exactly how the features in the model affect their judgments on disease classification.

In this study, we investigated the ability of ML models based on blood metabolites, AA, and AcylCN, to predict the DR risk in patients with T2D in northern China. Meanwhile, the best prediction model was explained by Shapley Additive exPlanation (SHAP) to quantify the effects of metabolites.

## 2. Materials and Methods

### 2.1. Research Design and Study Patients

Details of the study population and methods were described previously [13]. A total of 1898 T2D inpatients in Liaoning Medical University First Affiliated Hospital (LMUFAH) from May 2015 to August 2016 were enrolled. Diagnosis criteria for T2D were the 1999 WHO's criteria [14] or treated with antidiabetic agent. For these patients, we retrieved their electronic medical records and measured their AA and AcylCN profiles. Excluding missing age, AA, and AcylCN information, a total of 1032 patients aged 18 years or older were included in the current analysis.

### 2.2. Data Collection and Definitions

Electronic medical records provided the information on demographic, anthropometric, current status of smoking and alcohol drinking, duration of T2D, clinical and laboratory measurements, medicant used, and DR status. The clinical parameters included systolic blood pressure (SBP), diastolic blood pressure (DBP), triglyceride (TG), high-density lipoprotein (HDL) cholesterol, low-density lipoprotein (LDL) cholesterol, total cholesterol (TC), glycosylated hemoglobin (HbA1c), and serum creatinine (SCr). Medication information included antidiabetic agents, antihypertensive drugs, and lipid-lowering drugs.

### 2.3. Clinical Definitions

Body mass index (BMI) was calculated as weight in kilograms divided by height in meters squared (kg/m^2^) [15]. For Chinese people, BMI ≤ 18.5, 24-27.9, and ≥28 (kg/m^2^) are the appropriate cut-off points for underweight, overweight, and obesity [16]. Hyperglycemia was defined as HbA1c ≥ 7%. The definitions of abnormal lipids were HDL cholesterol < 1.0 mmol/L in men and HDL cholesterol < 1.3 mmol/L in women and/or LDL − C ≥ 2.6 mmol/L and/or TG ≥ 1.7 mmol/L [17]. DR was evaluated by the bilateral retinal photography and was defined as the presence, if any, of the following lesions: microaneurysms, retinal hemorrhages, soft exudates, hard exudates, or vitreous hemorrhage [9].

### 2.4. Laboratory Assays

Details of metabolomic approach for AA and AcylCN were published previously [18]. Briefly, dry blood spot (DBS) samples of patients were collected after 8 hours of fasting. The sample preparation process involved punching wells from DBS paper, adding working and quality control (QC) solutions, derivatizing and drying, and finally, dissolving the dried sample in fresh mobile phase solution. An AB Sciex 4000 QTrap system (AB Sciex, Framingham, MA, USA) was used to carry out the mass spectrometry metabolomic analysis. The ion source was electrospray ionization source. Analyst v1.6.0 software (AB Sciex) was used for system control and data collection. Isotope-labeled internal standards of AA (NSK-A) and AcylCN (NSK-B) from Cambridge Isotope Laboratories (Tewksbury, MA, USA) were used for preparing working solutions. AAs and carnitine QC standards were provided by Chromsystems (Grafelfing, Germany). Acetonitrile (high-performance liquid chromatography grade) was obtained from Thermo Fisher (Waltham, MA, USA).

### 2.5. Statistical Analysis

Continuous variables were reported as means with standard deviation (SD) or medians with interquartile ranges (IQRs), and categorical variables were reported as frequencies (%). Nonpaired Student's *t*-test, Mann–Whitney *U* test, and Chi-square tests (or Fisher's test, if appropriate) were conducted to estimate the differences of continuous and categorical data between patients with DR and not groups, respectively.

#### 2.5.1. Data Preparation

Data preparation was done as following. Categorical variables were dummy encoded (one binary variable for each category), and numerical variables with >30% of missing values were replaced with missing value indicators, which specify whether a value was missing (1) or not (0). Other features containing missing values were processed using multiple interpolations. Multiple imputations are implemented through the mouse package in R, with the number of imputations being 5.

#### 2.5.2. Feature Selection

Feature selection was performed by using least absolute shrinkage and selection operator (LASSO) regression. LASSO regression contains a regularization/penalty term in its cost function to prevent overfitting and ensure that the LASSO model neglects correlated features. According to the one standard error rule (1SE rule), the optimal value corresponds to the simplest model, and the cross-validation error of which is no more than one standard error above the minimum [19]. Therefore, we refer to the shrinkage parameter determined using the 1SE rule as the optimal value to obtain least features in this study.

#### 2.5.3. Model Training and Validation

Construction of ML prediction models for DR risk incorporated LASSO postscreening features and traditional clinical features, respectively. Logistic regression (LR) and extreme gradient boosting (XGBoost) algorithms were selected as ML classifiers in this study. The dataset was split into a training set and testing set at a ratio of 7 : 3 randomly. The best trained model was obtained in the training set using grid search. Moreover, we used the area under the curve (AUC), respectively, of the receiver operating characteristic (ROC) curve and precision recall (PR) curve analysis to evaluate its discrimination. The calibration curve and the Brier score were used to evaluate the calibration. The closer the calibration curve is to the reference line and the smaller the Brier score, the better the predictive calibration of the model. These evaluations were performed in the testing set.

#### 2.5.4. Model Interpretation

The effects of features on prediction scores were measured by SHAP, which assessed the importance of each feature using a game-theoretic approach based on the testing set [20]. The SHAP value quantifies the marginal contribution of each feature to the final prediction, which in our case is a T2D patient developing DR or not. In other words, SHAP value is the contribution of a given feature *i* in the prediction model and is the difference between the prediction using the value of *i* and the mean prediction [21].

*P* values of <0.05 were considered statistically significant in all these analyses. Data preparation and feature selection were conducted using R V.4.1.1. Modeling, evaluation, and interpretation of ML models were conducted using Python V.3.10. The whole technical details and machine learning pipeline are given in supplementary figure 1.

## 3. Result

### 3.1. Characteristics of the Study Patients

The 1032 patients had a mean age of 57.2 (SD 13.8) years and a median duration of T2D of 5 (IQR 0–10) years. Of these patients, 53.2% were male. The mean BMI of the cohort was 25.3 (SD 3.9) kg/m^2^. Of these patients, 162 were with prior DR, while 870 were not. Subjects with DR composed more women and had longer duration of diabetes and higher SBP and TC. Age, BMI, DBP, current smoking and drinking, HbA1c, creatinine, and drug use were similar in the two groups (Table 1).

We observed 11 AAs and 19 AcylCNs demonstrating lower levels (*P* < 0.05) in T2D patients with DR. Other AAs and AcylCNs were similar in the two groups (see Supplementary table 1 & table 2).

### 3.2. Feature Selection

**Figure 1:**
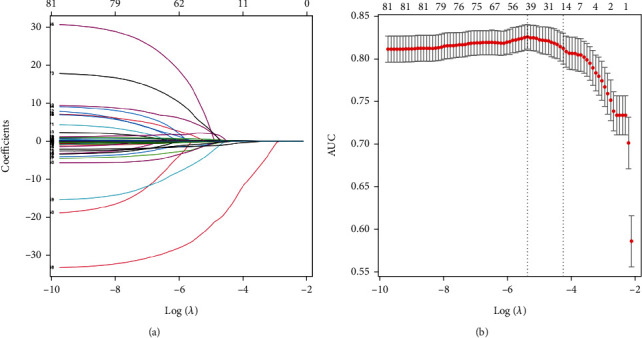
(a) LASSO coefficient profiles of the 82 features. (b) Tuning parameter (*λ*) selection in the LASSO model used 10-fold cross-validation via minimum criteria. The area under the receiver operating characteristic curve (AUC) was plotted versus log(*λ*). Dotted vertical lines were drawn at the optimal values by using the minimum criteria and the 1SE criteria. 14 features with nonzero coefficients were selected according to the 1SE criterion.

After LASSO regression screening, the number of features was reduced from the initial 82 to 15 (Figure 1). Metabolite features included 7 AAs (alanine, citrulline, glutamate, ornithine, phenylalanine, threonine, and tyrosine) and 3 AcylCNs (octacarbonylcarnitine (C18 : 1), 3-hydroxy-octadecylcarnitine (C18 : 1OH), and octadecadienylcarnitine (C18 : 2)). Additionally, patients' age, SBP, TC, duration of T2D, and missing value indicator of HbA1c have also been contained. Considering that the missing value indicator is not clinically significant for the disease, it was not included in the follow-up modeling.

### 3.3. Model Training and Validation

**Figure 2:**
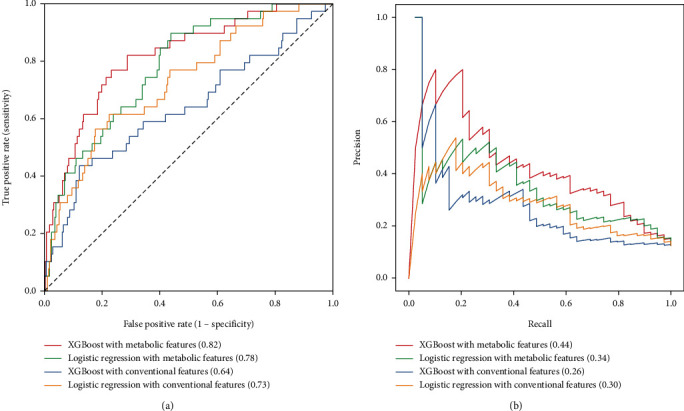
(a) Receiver operating characteristic curves of predictive models. Red line means XGBoost model with LASSO selection features (AUC = 0.82), green line means logistic regression model with LASSO selection features (AUC = 0.78), blue line means XGBoost model with conventional features (AUC = 0.64), and yellow line means logistic regression model with conventional features (AUC = 0.73). (b) Precision recall curves of predictive models. Red line means XGBoost model with LASSO selection features (AUC = 0.44), green line means logistic regression model with LASSO selection features (AUC = 0.34), blue line means XGBoost model with conventional features (AUC = 0.26), and yellow line means logistic regression model with conventional features (AUC = 0.30).

Four ML models were eventually constructed, LR model 1 and XGBoost model 1 using traditional clinical features and LR model 2 and XGBoost model 2 using LASSO selection features. The discrimination of each model was assessed by AUC (Table 2 and Figure 2). Among them, the ROCAUC of XGBoost model 2 in the testing set was 0.82 (95% CI, 0.75-0.82), increasing significantly 9% than LR model 1 (ROCAUC, 0.73; 95% CI, 0.68-0.74; *P* = 0.006). Considering the imbalance of patient dataset, we used the AUC of PR curve as a secondary evaluation metric for differentiation. Results showed that there was still room for improvement in these models, but XGBoost model 2 still performed best (PRAUC, 0.44; 95% CI, 0.31-0.47).

**Figure 3:**
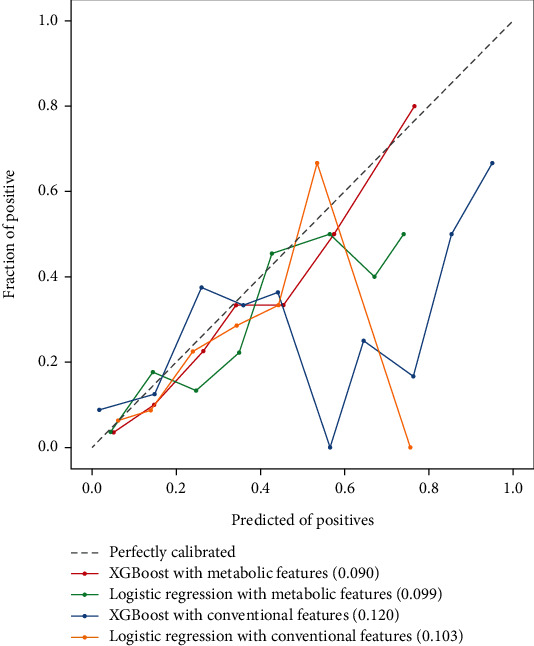
The calibration curve of predictive models. Red line means XGBoost model with LASSO selection features (Brier score = 0.090), green line means Logistic Regression model with LASSO selection features (Brier score = 0.099), blue line means XGBoost model with conventional features (Brier score = 0.120), yellow line means Logistic Regression model with conventional features (Brier score = 0.103), grey dotted line means perfectly calibrated.

The evaluation of calibration in testing set have been shown in Figure 3 that XGBoost model 2 demonstrated the best agreement between prediction and observation. The Brier score of XGBoost model 2 was 0.09.

### 3.4. Model Interpretation

**Figure 4:**
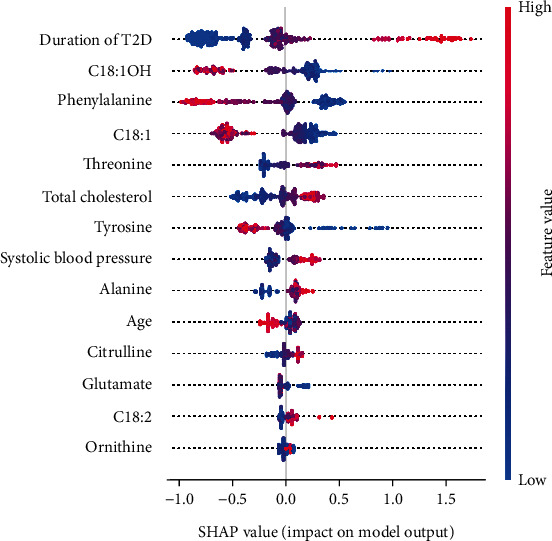
SHAP summary plot of XGBoost model with LASSO selection features (XGBoost model 2). Each point on the summary plot is a Shapley value for a feature and an instance. The position on the *y*-axis is determined by the feature and on the *x*-axis by the Shapley value. The color represents the value of the feature from low to high. The features are ordered according to their importance. T2D: type 2 diabetes; C18 : 1OH: 3-hydroxy-octadecylcarnitine; C18 : 1: octacarbonylcarnitine; C18 : 2: octadecadienylcarnitine; SHAP: Shapley Additive exPlanation.

A SHAP summary plot of XGBoost model 2 showed the importance of features (Figure 4). When the Shapley value of each feature exceeds zero, it indicates an increased risk of DR. The scatter colors in the graph reflect the magnitude of the eigenvalues (larger in red, smaller in blue). As shown in Figure 4, the duration of T2D, C18 : 1OH, phenylalanine, C18 : 1, threonine, TC, and tyrosine contributed more to the model. Furthermore, the high value of duration of T2D, threonine, and TC and low value of C18 : 1OH, phenylalanine, C18 : 1, and tyrosine corresponded to a Shapley value greater than zero. This suggested that these features were important risk factors to DR.

**Figure 5:**
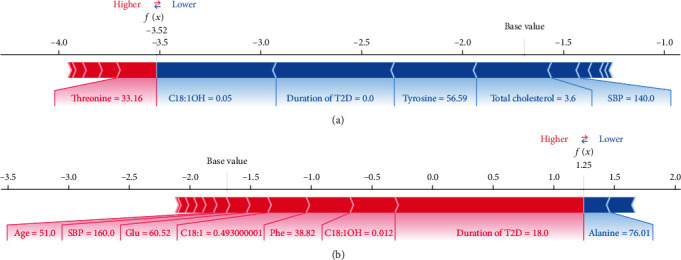
Two examples of the local explanation of the predictions using the Shapley Additive exPlanation (SHAP) values. (a) Predicted type 2 diabetes patient without diabetic retinopathy. (b) Predicted type 2 diabetes patient with diabetic retinopathy. Factors that push the predicted score higher compared to the base value (mean prediction) are coloured red, and those pushing lower the prediction are shown in blue. C18 : 1OH: 3-hydroxy-octadecylcarnitine; T2D: type 2 diabetes; SBP: systolic blood pressure; Glu: glutamate; C18 : 1: octacarbonylcarnitine; Phe: phenylalanine; C18 : 2: octadecadienylcarnitine.

When most features are normal and for new-onset diabetes patients, the risk of developing DR is low (Figure 5(a)). When the duration of T2D is longer and most features (C18 : 1OH, phenylalanine, C18 : 1, glutamate, and SBP) are abnormal, the risk of DR increases (Figure 5(b)).

**Figure 6:**
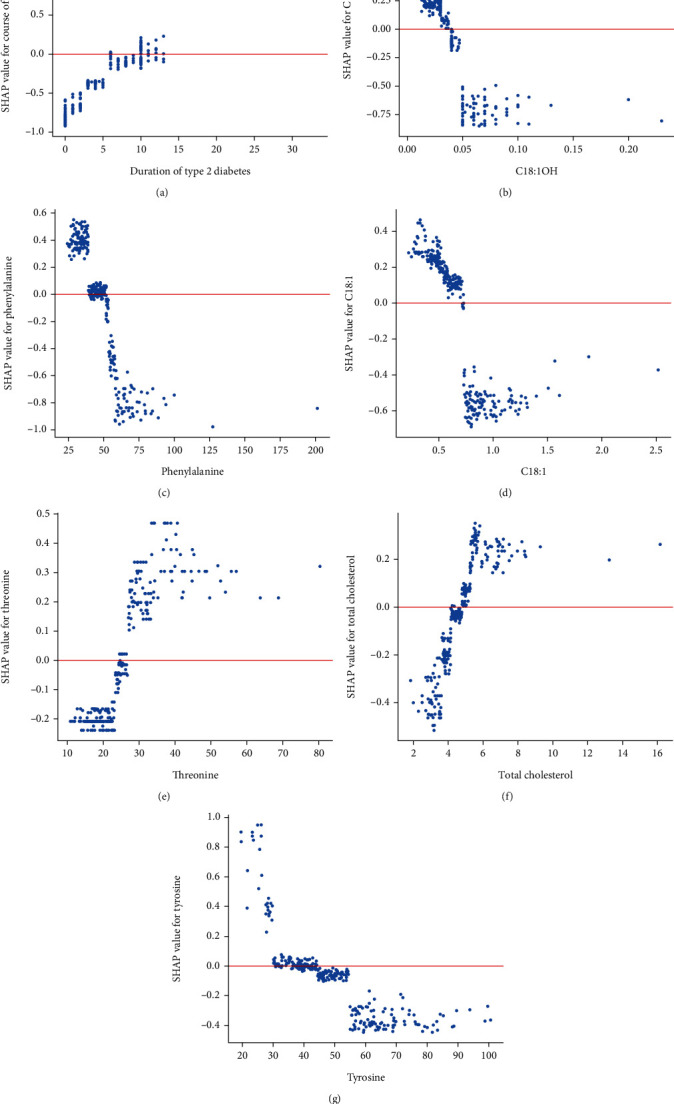
SHAP dependence plots of important features in XGBoost model. (a) SHAP dependence plots of duration of type 2 diabetes; (b) SHAP dependence plots of C18 : 1OH; (c) SHAP dependence plots of phenylalanine; (d) SHAP dependence plots of C18 : 1; (e) SHAP dependence plots of threonine; (f) SHAP dependence plots of total cholesterol; (g) SHAP dependence plots of tyrosine. The blue dots represent the eigenvalues and the Shapley values corresponding to each observation. The red line represents the Shapley values equal to zero. When the Shapely value corresponding to a characteristic was greater than zero, the risk of developing DR is considered to be increased under that condition. C18 : 1OH: 3-hydroxy-octadecylcarnitine; C18 : 1; octacarbonylcarnitine; C18 : 2: octadecadienylcarnitine; SHAP: Shapley Additive exPlanation.

To further explain the impact of each risk factor on the DR forecast, we have created the SHAP dependence plots (Figure 6). These features all showed clearer cut-off values. Duration of T2D more than 10 years, C18 : 1OH lower than 0.04 *μ*mol/L, C18 : 1 lower than 0.70 *μ*mol/L, threonine higher than 27.0 *μ*mol/L, TC higher than 4.75 mmol/L, and tyrosine lower than 36.0 *μ*mol/L is associated with increased risk of developing DR. When patients' blood level of phenylalanine is higher than 52.0 *μ*mol/L, it might be associated with a decreased risk of developing DR.

## 4. Discussion

In this study, a machine learning DR risk model with good predictive ability was constructed, and the effect of the predictive features on DR was analyzed. The results showed that metabolomic features such as C18 : 1OH, phenylalanine, C18 : 1, threonine, and tyrosine played a great role in the prediction model.

In the early stage of DR, there are no obviously clinical symptoms and signs occurred, but pathophysiological changes are quietly progressing [22]. When the disease progresses to an advanced stage, the resulting lesions are irreversible. Therefore, early detection of high-risk group of DR and providing timely fundus screening for patients are important. Glycemia played a central role in the development and progression of microvascular complications of diabetes. But studies had shown that DR still progressed under good glycemic control in patients [23, 24]. Therefore, it is essential to explore new biomarkers and develop disease prediction models. Dagliati et al. used electronic medical record data to construct ML prediction models for diabetic complications [25]. Their study demonstrated that ML algorithms were powerful tools for clinical model development and medical data mining. However, electronic medical record data contains features from a wide range of sources, making it difficult to collect and prone to missing data. In the present study, the features incorporated into the model were simplified, relying primarily on AAs and AcylCNs for prediction of disease. Not only it is easy to implement, but it identifies the early stages of disease.

The pathogenesis of DR is complex, involving a variety of endothelial dysfunction, inflammation, oxidative stress, and neural mechanisms [22, 26]. In addition, DR patients also have the characteristics of disorders of glucose and lipid metabolism of patients with T2D. AAs and AcylCNs that have been incorporated into the ML model are likely to be involved in these pathogenic mechanisms.

### 4.1. Acylcarnitine in Type 2 Diabetes and Diabetic Retinopathy

Fatty acid oxidation (FAO) defect and metabolic derangements, such as insulin resistance, could be screened by the mass spectrometric analysis of carnitine profile [27]. There are some studies shown that levels and abundance of several long-chain AcylCNs were significantly decreased in T2D and DR patients. Reasonable speculation for results was an inhibited carnitine palmitoyltransferase-1- (CPT1-) mediated entry of free fatty acid (FA) into mitochondria and impaired mitochondrial *β*-oxidation of retinal FA in the retina [28–30]. From this perspective, reduced levels of long-chain AcylCN might be considered as a biomarker for metabolic abnormalities or a risk factor for disease, which was consistent with our results. However, there were also inconsistent studies showing a higher level of AcylCN in T2D, which might due to incomplete oxidation of long-chain FA and altered tricarboxylic acid cycle (TCA) activity in patients with T2D [31].

Dyslipidemia and lip toxicity (accumulation of lipid metabolites) are increasingly recognized as important drivers of insulin resistance states [32]. Therefore, another focus of AcylCN in T2D is whether AcylCN via the impairments of FA oxidation reflect or inflict insulin resistance [27]. In the mitochondrial lipid metabolism, AcylCN not only prevents the accumulation of noxious acyl coenzyme A (CoA) but also reduces CoA trapping. It means that AcylCN formation allows continuation of CoA-dependent metabolic, such as the carnitine shuttle and FAO, processes [33]. In animal experiments, incomplete muscle FA *β*-oxidation leads to AcylCN accumulation and associated oxidative stress, which may be responsible for the development of muscle insulin resistance [34].

Due to the insufficiency of reports about AcylCN in the DR, current inferences were mainly based on T2D. For the unclear pathogenesis of the disease, whether these theories can be applied in the pathogenesis of DR requires further study. In addition, the wide variation in study populations (sex, ethnicity, genetics, and sample size) might have led to inconsistent results.

### 4.2. Amino Acid in Type 2 Diabetes and Diabetic Retinopathy

Low phenylalanine and low tyrosine are important factors in our model. Tyrosine and phenylalanine as the preferred and secondary precursors of dopamine (DA) which rates of synthesis were influenced by local substrate concentrations and dysregulation were contributed to retinal neurodegeneration [35–37]. In our study, tyrosine lower than 36.0 *μ*mol/L and phenylalanine lower than 52.0 *μ*mol/L increased the risk of DR, which might be related to the mechanism mentioned above. Impaired L-threonine catabolism has been shown to promote methylglyoxal (MGO) accumulation [38]. MGO is a highly reactive aldehyde which associates to impairment of regulatory mechanisms of retinal blood flow and hyperpermeability of the blood-retinal barrier in DR [39]. This was consistent with the results of our study.

Besides AAs and AcylCNs, four factors—duration of T2D, TC, SBP and age—were included in the XGBoost model in this study. Of these, the duration of T2D had the greatest impact on the model classification and increased the risk of DR when the T2D lasts more than 10 years. Other studies and literature reviews also considered the duration of diabetes as an important risk factor for DR [33].

The inconsistency with some studies was shown in our model interpretation, and part of the reasons has been stated earlier. However, what cannot be ignored is that the SHAP summary plots describe the behavior of the (imperfect) predictive model and not necessarily the causal relationships between the variables [40].

### 4.3. Strengths and Limitations

Because of the late onset of DR symptoms, the difficulty in detecting them, and the high demand for physicians and equipment for funduscopic examinations, diagnostic prescriptions are rarely issued when patients are asymptomatic. Our prediction model could remind doctors and patients to pay attention to the primary and secondary prevention of DR and increase the fundus screening rate of the high-risk groups. Secondly, our study again suggested that lipid and AA metabolism may have an important role in the development of DR. The combination of AA and AcylCN has the potential to be a new predictive marker of disease. Finally, with the SHAP interpretation, the features incorporated in our ML model are relatively homogenous in origin and easily accessible. This provides the basis for the development of predictive models for clinical application.

Our study also has the following limitations. First, this study was based on a cross-sectional study of inpatients and cannot infer the causal relationship between accumulation of AA and AcylCN and DR. Second, HbA1c was not included in this study due to its excessive deficiency. Third, this study did not evaluate the effects of specific drugs, such as metformin and sodium-glucose cotransporter 2 (SGLT2). But we considered the overall influence of antidiabetic agents, antihypertensive drugs, and lipid-lowering drugs. Almost T2D patients have received treatment which might result in these features not being involved in the model. Moreover, this is a single-center study without independent external validation. In the future, we will try to develop models in larger scale data and explore the associations between metabolites with DR in prospective study.

## 5. Conclusions

In conclusion, our study mainly used AAs and AcylCNs to construct an interpretable XGBoost model to predict the risk of developing DR in patients with T2D. The aim is to identify high-risk groups and then to improve fundus screening rates in high-risk groups and reduce the burden of disease. In addition, our study proposed possible risk cut-off values for DR for C18 : 1OH, C18 : 1, threonine, tyrosine, and phenylalanine. The study is beneficial in preventing or delaying the onset of DR. In the future, larger prospective studies will be needed to validate this result.

## Figures and Tables

**Table 1:** Characteristics of patients with T2D according to DR status.

	Total^a^	DR^b^	No-DR^c^	*P* value^d^
*n*	1032	162	870	
Age, years	57 (14)	58 (10)	57 (14)	.491
Sex, male	549 (53.2%)	73 (45.1%)	476 (54.7%)	.030
Duration of T2D, years	5 (0-10)	13 (6-20)	4 (0-10)	<.001
Body mass index, kg/m^2^	25.3 (3.9)	25.1 (3.3)	25.3 (4.0)	.368
~18.5	27 (2.6%)	1 (0.6%)	26 (3.0%)	.162
18.5 ~ 23.9	352 (34.1%)	64 (39.5%)	288 (33.1%)	
24.0 ~ 27.9	430 (41.7%)	66 (40.7%)	364 (41.8%)	
28.0 ~	223 (21.6%)	31 (19.1%)	192 (22.1%)	
Systolic blood pressure, mmHg		145.6 (25.2)	139.5 (23.6)	.004
Diastolic blood pressure, mmHg		82.0 (73.3-90.0)	81.0 (74.0-90.0)	.647
Glycosylated hemoglobin, %		9.5 (7.4-11.0)	9.6 (7.8-11.1)	.380
<7		15 (15.8%)	62 (11.6%)	.323
≥7		80 (84.2%)	474 (88.4%)	
Missing		67	334	
Triglyceride, mmol/L		1.72 (1.14-2.46)	1.66 (1.11-2.37)	.544
<1.70		46 (48.9%)	337 (51.8%)	.676
≥1.70		48 (51.1%)	313 (48.2%)	
Missing		68	220	
High-density lipoprotein cholesterol, mmol/L		1.04 (0.87-1.31)	1.01 (0.85-1.25)	.254
<1.00 in men or <1.30 in women		57 (60.6%)	429 (66.3%)	.335
≥1.00 in men or ≥1.30 in women		37 (39.4%)	218 (33.7%)	
Missing		68	223	
Low-density lipoprotein cholesterol, mmol/L		2.83 (2.27-3.36)	2.78 (2.20-3.38)	.583
<2.60		269 (41.6%)	38 (40.4%)	.921
≥2.60		378 (58.4%)	56 (59.6%)	
Missing		68	223	
Total cholesterol, mmol/L		4.84 (4.00-5.66)	4.63 (3.83-5.27)	.043
Serum creatinine, *μ*mol/L		56.65 (48.77-72.49)	60.22 (49.39-74.49)	.314
Current smoking		42 (25.9%)	289 (33.2%)	.083
Current drinking		42 (25.9%)	248 (28.5%)	.565
Antidiabetic agents		137 (84.6%)	730 (83.9%)	.925
Lipid-lowering agents		63 (38.9%)	325 (37.4%)	.778
Hypotensive agents		86 (53.1%)	457 (52.5%)	.964

Notes: data are mean (standard deviation), median (IQR), or *n* (%). ^a^All subjects were analyzed for age, sex, duration of type 2 diabetes, body mass index, and body mass index categories. ^b^Type 2 diabetic patients with retinopathy. ^c^Type 2 diabetic patients without retinopathy. ^d^*P* values were derived from independent sample Student's *t*-test for normally distributed variables, Mann–Whitney *U* test for skewed distributions, and Chi-square test (or Fisher's test if appropriate) for categorical variables. *P* < 0.05 was defined as statistically significant. Abbreviations: T2D: type 2 diabetes; DR: diabetic retinopathy.

**Table 2:** Discriminant evaluation of predictive models.

Model	Accuracy	ROCAUC (95% CI)	*P* value^a^	PRAUC (95% CI)	*P* value^b^
LR model 1	87.42%	0.73 (0.68, 0.74)	Ref	0.30 (0.24, 0.32)	Ref
XGBoost model 1	83.87%	0.64 (0.61, 0.72)	.023	0.26 (0.21, 0.39)	.312
LR model 2	87.10%	0.78 (0.73, 0.81)	.156	0.34 (0.27, 0.40)	.283
XGBoost model 2	88.39%	0.82 (0.75, 0.82)	.006	0.44 (0.31, 0.47)	<.001

Notes: features in model 1: sex, age, duration of type 2 diabetes, body mass index, systolic blood pressure, diastolic blood pressure, triglyceride, high-density lipoprotein cholesterol, low-density lipoprotein cholesterol, and total cholesterol. Features in model 2: duration of type 2 diabetes, age, systolic blood pressure, total cholesterol, alanine, citrulline, glutamate, ornithine, phenylalanine, threonine, tyrosine, C18 : 1, C18 : 1OH, and C18 : 2. ^a^Delong test for area under the curve of receiver operating characteristic curve. ^b^Delong test for area under the curve of precision recall curve. Abbreviations: ROC: receiver operating characteristic; AUC: area under the curve; CI; confidence interval; PR: precision recall; LR: logistic regression; XGBoost: extreme gradient boosting; Ref: reference.

## Data Availability

Data are available upon reasonable request. Our study collected clinical information from the electronic medical systems retrospectively. Our data are not in a repository. If someone is interested in our data, please contact us via email (fangzhongze@tmu.edu.cn). One should include his detailed statistical analysis plans in his email.

## Supplementary Materials

Supplementary Materials Flow charts for data analysis and complex long table results are provided in the supplementary material. Supplementary table 1: amino acids of patients with T2D according to DR status. Supplementary table 2: acylcarnitines of patients with T2D according to DR status. Supplementary figure 1: participant flow chart and machine learning pipeline. Supplementary figure 2: feature selection results of LASSO regression. Supplementary figure 3: receiver operating characteristic curves of XGBoost model with LASSO selection features. Supplementary figure 4: SHAP summary plot of XGBoost model with LASSO selection features after attribute scaling.
