# Supplementary material for: A Metabolism-Based Interpretable Machine Learning Prediction Model for Diabetic Retinopathy Risk: A Cross-Sectional Study in Chinese Patients with Type 2 Diabetes
Source: J Diabetes Res. 2023 May 16;2023:3990035. doi: 10.1155/2023/3990035 (PMC10205414; doi:10.1155/2023/3990035)
Supplement: Supplementary Materials — Flow charts for data analysis and complex long table results are provided in the supplementary material. Supplementary table 1: amino acids of patients with T2D according to DR status. Supplementary table 2: acylcarnitines of patients with T2D according to DR status. Supplementary figure 1: participant flow chart and machine learning pipeline. Supplementary figure 2: feature selection results of LASSO regression. Supplementary figure 3: receiver operating characteristic curves of XGBoost model with LASSO selection features. Supplementary figure 4: SHAP summary plot of XGBoost model with LASSO selection features after attribute scaling. [file 3990035.f1.docx]

# A metabolism-based interpretable machine learning prediction model for diabetic retinopathy risk: a cross-sectional study in Chinese patients with type 2 diabetes

**
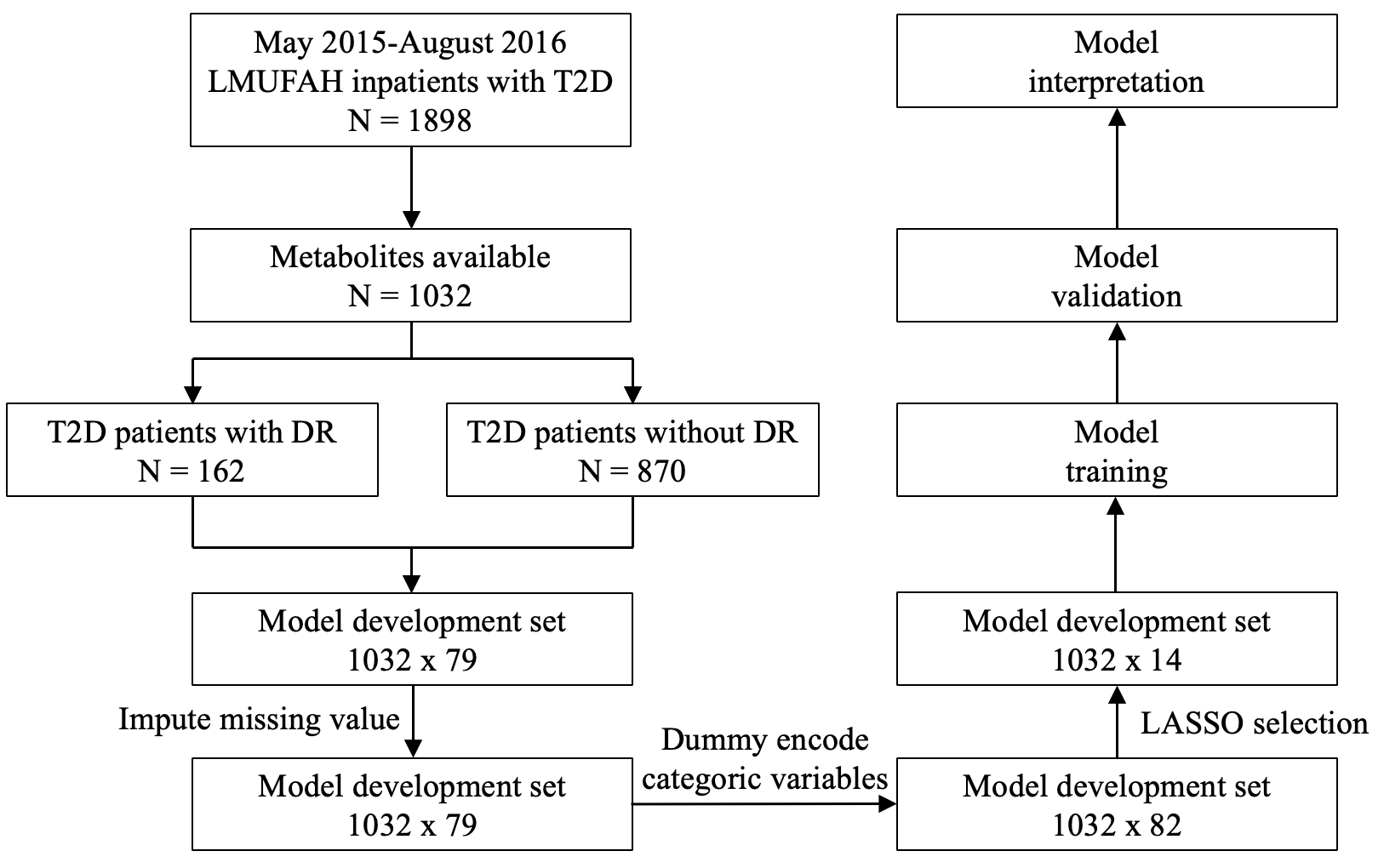
**

Supplementary figure 1 Participant flow chart and machine learning pipeline.

LMUFAH, Liaoning Medical University First Affiliated Hospital; T2D, type 2 diabetes; DR, diabetic retinopathy; LASSO, least absolute shrinkage and selection operator.

| Supplementary table 1: Amino acids of patients with T2D according to DR status | | | |
| --- | --- | --- | --- |
| **Metabolites, μmol/L** | **DR ^a^** | **no-DR ^b^** | **P value ^c^** |
| Alanine | 126.63 (102.45-148.20) | 124.75 (98.40-154.72) | 0.951 |
| Arginine | 10.23 (6.34-16.26) | 10.64 (5.77-17.40) | 0.874 |
| Asparagine | 72.02 (58.08-85.45) | 75.53 (62.40-90.60) | 0.008 |
| Aspartate | 27.12 (21.04-35.83) | 28.74 (20.93-37.88) | 0.287 |
| Citrulline | 21.20 (16.36-27.35) | 19.63 (15.33-25.68) | 0.099 |
| Cysteine | 1.33 (0.59) | 1.56 (5.48) | 0.226 |
| Glutarnine | 7.10 (5.46-9.63) | 6.78 (5.01-9.20) | 0.104 |
| Glutamate | 91.94 (72.41-110.63) | 99.51 (82.00-121.98) | <0.001 |
| Glycine | 177.97 (135.59-231.65) | 206.68 (155.02-269.63) | <0.001 |
| Homocysteine | 7.88 (6.57-8.56) | 7.88 (6.57-8.37) | 0.620 |
| Histidine | 44.01 (34.00-67.22) | 52.74 (35.77-79.80) | 0.007 |
| Leucine | 115.86 (93.48-137.04) | 127.81 (102.06-159.39) | <0.001 |
| Lysine | 147.27 (68.63) | 140.27 (79.04) | 0.246 |
| Methionine | 16.59 (13.33-20.60) | 17.26 (14.46-21.42) | 0.031 |
| Ornithine | 16.02 (11.81-23.13) | 17.59 (13.36-23.73) | 0.158 |
| Phenylalanine | 39.00 (33.82-48.00) | 46.33 (38.16-56.46) | <0.001 |
| Piperidine | 126.71 (92.01-163.17) | 127.98 (94.93-175.87) | 0.494 |
| Proline | 423.77 (331.68-563.14) | 445.25 (337.85-598.51) | 0.267 |
| Serine | 48.17 (41.54-58.67) | 52.14 (43.81-65.24) | 0.001 |
| Threonine | 24.29 (18.99-30.41) | 24.52 (19.77-30.53) | 0.419 |
| Tryptophan | 43.91 (36.13-51.95) | 47.97 (39.08-57.06) | <0.001 |
| Tyrosine | 39.52 (31.69-49.90) | 46.45 (37.85-57.68) | <0.001 |
| Valine | 132.24 (110.88-155.29) | 137.55 (114.15-163.91) | 0.037 |

Notes: Data is median (interquartile range).

a Type 2 diabetic patients with retinopathy.

b Type 2 diabetic patients without retinopathy.

d P values were derived from independent-samples Student t test for normally distributed variables, Mann-Whitney U test for skewed distributions. P < 0.05 was defined as statistically significant.

Abbreviations: T2D, type 2 diabetes; DR, diabetic retinopathy.

| Supplementary table 2: Acylcarnitines of patients with T2D according to DR status | | | |
| --- | --- | --- | --- |
| **Metabolites, μmol/L** | **DR ^a^** | **no-DR ^b^** | **P value ^c^** |
| C0 | 26.06 (20.76-32.39) | 27.91 (21.36-35.33) | 0.064 |
| C2 | 10.44 (8.17-12.90) | 11.97 (8.99-15.85) | <0.001 |
| C3 | 1.18 (0.84-1.49) | 1.41 (0.97-2.00) | <0.001 |
| C3DC | 0.09 (0.06-0.13) | 0.09 (0.07-0.12) | 0.754 |
| C4 | 0.19 (0.15-0.25) | 0.21 (0.15-0.28) | 0.036 |
| C4OH | 0.09 (0.07-0.13) | 0.11 (0.08-0.16) | <0.001 |
| C4DC | 0.63 (0.47-0.81) | 0.65 (0.49-0.86) | 0.381 |
| C5 | 0.12 (0.09-0.17) | 0.15 (0.11-0.20) | <0.001 |
| C5OH | 0.27 (0.12) | 0.29 (0.13) | 0.078 |
| C5DC | 0.07 (0.05-0.12) | 0.08 (0.05-0.12) | 0.145 |
| C5:1 | 0.06 (0.04-0.08) | 0.06 (0.05-0.08) | 0.215 |
| C6 | 0.05 (0.03-0.06) | 0.05 (0.04-0.07) | 0.051 |
| C6DC | 0.75 (0.51-0.99) | 0.81 (0.62-1.08) | 0.009 |
| C8 | 0.06 (0.04-0.10) | 0.07 (0.05-0.10) | 0.691 |
| C10 | 0.09 (0.06-0.16) | 0.09 (0.06-0.15) | 0.668 |
| C10:1 | 0.11 (0.07-0.19) | 0.11 (0.08-0.16) | 0.930 |
| C10:2 | 0.73 (0.49-1.11) | 0.77 (0.54-1.06) | 0.723 |
| C12 | 0.05 (0.04-0.07) | 0.05 (0.04-0.07) | 0.247 |
| C14 | 0.06 (0.05-0.08) | 0.07 (0.05-0.09) | <0.001 |
| C14OH | 0.05 (0.04-0.06) | 0.05 (0.04-0.07) | 0.121 |
| C14DC | 0.04 (0.02) | 0.05 (0.03) | <0.001 |
| C14:1 | 0.09 (0.06-0.12) | 0.10 (0.07-0.13) | 0.011 |
| C14:2 | 0.71 (0.45-0.91) | 0.71 (0.51-0.99) | 0.245 |
| C16 | 0.80 (0.65-0.96) | 0.94 (0.74-1.20) | <0.001 |
| C16OH | 0.02 (0.02-0.03) | 0.03 (0.02-0.04) | 0.036 |
| C16:1OH | 0.05 (0.04-0.06) | 0.06 (0.04-0.07) | <0.001 |
| C18 | 0.41 (0.33-0.50) | 0.46 (0.36-0.58) | <0.001 |
| C18OH | 0.02 (0.02-0.03) | 0.03 (0.02-0.03) | 0.013 |
| C18:1 | 0.57 (0.48-0.68) | 0.65 (0.52-0.84) | <0.001 |
| C18:1OH | 0.03 (0.02-0.03) | 0.03 (0.02-0.05) | <0.001 |
| C18:2 | 1.66 (1.27-2.06) | 1.73 (1.28-2.18) | 0.260 |
| C20 | 0.04 (0.04-0.05) | 0.05 (0.04-0.06) | 0.028 |
| C22 | 0.07 (0.05-0.09) | 0.08 (0.06-0.10) | <0.001 |
| C24 | 0.05 (0.04-0.07) | 0.06 (0.04-0.07) | 0.192 |
| C26 | 0.03 (0.02-0.05) | 0.04 (0.03-0.05) | 0.029 |

Notes: Data is median (interquartile range).

a Type 2 diabetic patients with retinopathy.

b Type 2 diabetic patients without retinopathy.

d P values were derived from independent-samples Student t test for normally distributed variables, Mann-Whitney U test for skewed distributions. P < 0.05 was defined as statistically significant.

Abbreviations: T2D, type 2 diabetes; DR, diabetic retinopathy; C0, free carnitine; C2, acetylcarnitine; C3, propionylcarnitine; C3DC, propionylcarnitine; C4, butyrylcarnitine; C4DC, succinylcarnitine; C4OH, hydroxylbutyrylcarnitine; C5, isovalerylcarnitine; C5OH, 3-hydroxyisovalerylcarnitine; C5DC, glutarylcarnitine; C5:1, tiglylcarnitine; C6, hexanoylcarnitine; C6DC, adipylcarnitine; C8, octanoylcarnitine; C10, decanoylcarnitine; C10:1, decanoylcarnitine; C10:2, sebacoylcarnitine; C12, lauroylcarnitine; C14, myristoylcarnitine; C14OH, 3-hydroxyl- tetradecanoylcarnitine; C14DC, tetradecanoyldiacylcarnitine; C14:1, tetradecenoylcarnitine; C14:2, tetradecadienylcarnitine; C16, palmitoylcarnitine; C16OH, 3- hydroxypalmitoylcarnitine; C16:1OH, 3-hydroxypalmitoleylcarnitine; C18, octadecanoylcarnitine; C18OH, 3-hydroxy-octadecoylcarnitine; C18:1, octacarbonylcarnitine; C18:1OH, 3- hydroxy-octadecylcarnitine; C18:2, octadecadienylcarnitine; C20, arachidic carnitine; C22, behenic carnitine; C24, tetracosanoic carnitine; C26, hexacosanoic carnitine.

Sensitivity analysis results

Sensitivity analysis was performed to check the influence of with or without attribute scaling before LASSO regression in this study. Besides attribute scaling being performed before the feature selection, other steps of analysis were the same as the statistical analysis in the paper.


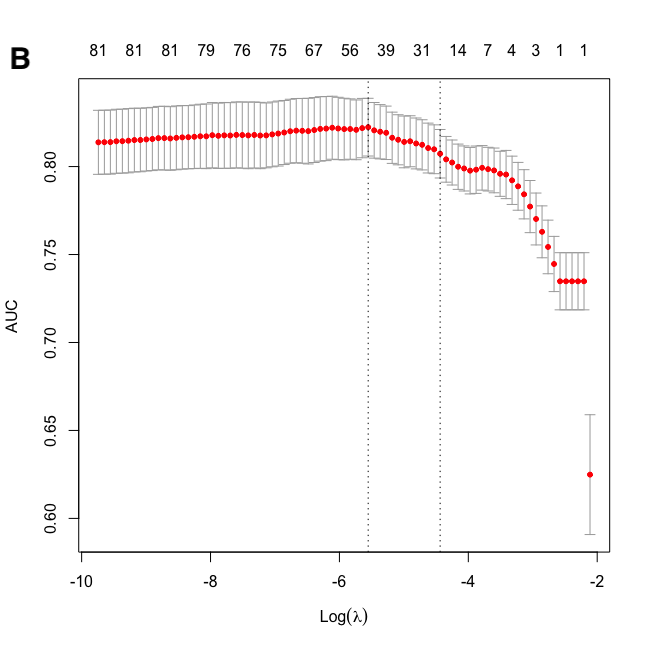

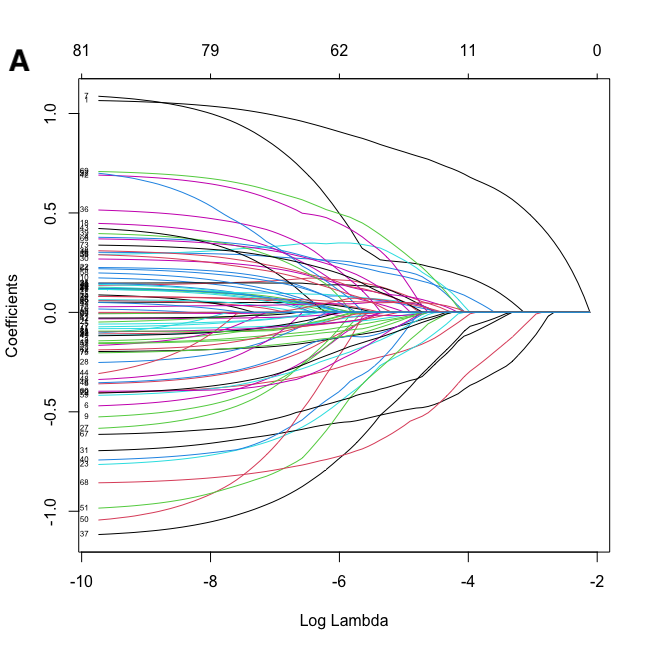
After attribute scaling and LASSO regression screening, the number of features was reduced from the initial 82 to 19 (Supplementary Fig 2). There were alanine, citrulline, glutamate, ornithine, phenylalanine, threonine, tyrosine, octacarbonylcarnitine (C18:1), 3-hydroxy-octadecylcarnitine (C18:1OH), octadecadienylcarnitine (C18:2), age, SBP, TC, duration of T2D and missing value indicator of HbA1 which have been list in the paper. Additionally, four new features were contained. There were hypoglycemic drug using, adipylcarnitine (C6DC), BMI<18.5 kg/m^2^, and 18.5≤BMI<24.0 kg/m^2^.

Supplementary figure 2 Feature selection results of LASSO regression

The AUC of the XGBoost model is 0.80 and still the highest of four models (Supplementary Fig 3). SHAP summary plot shows that the top 7 features did not change (Supplementary Fig 4). The four new features make little contribution to the prediction.

According to the sensitivity analysis results, there was not a significant influence on our conclusion before and after attribute scaling.


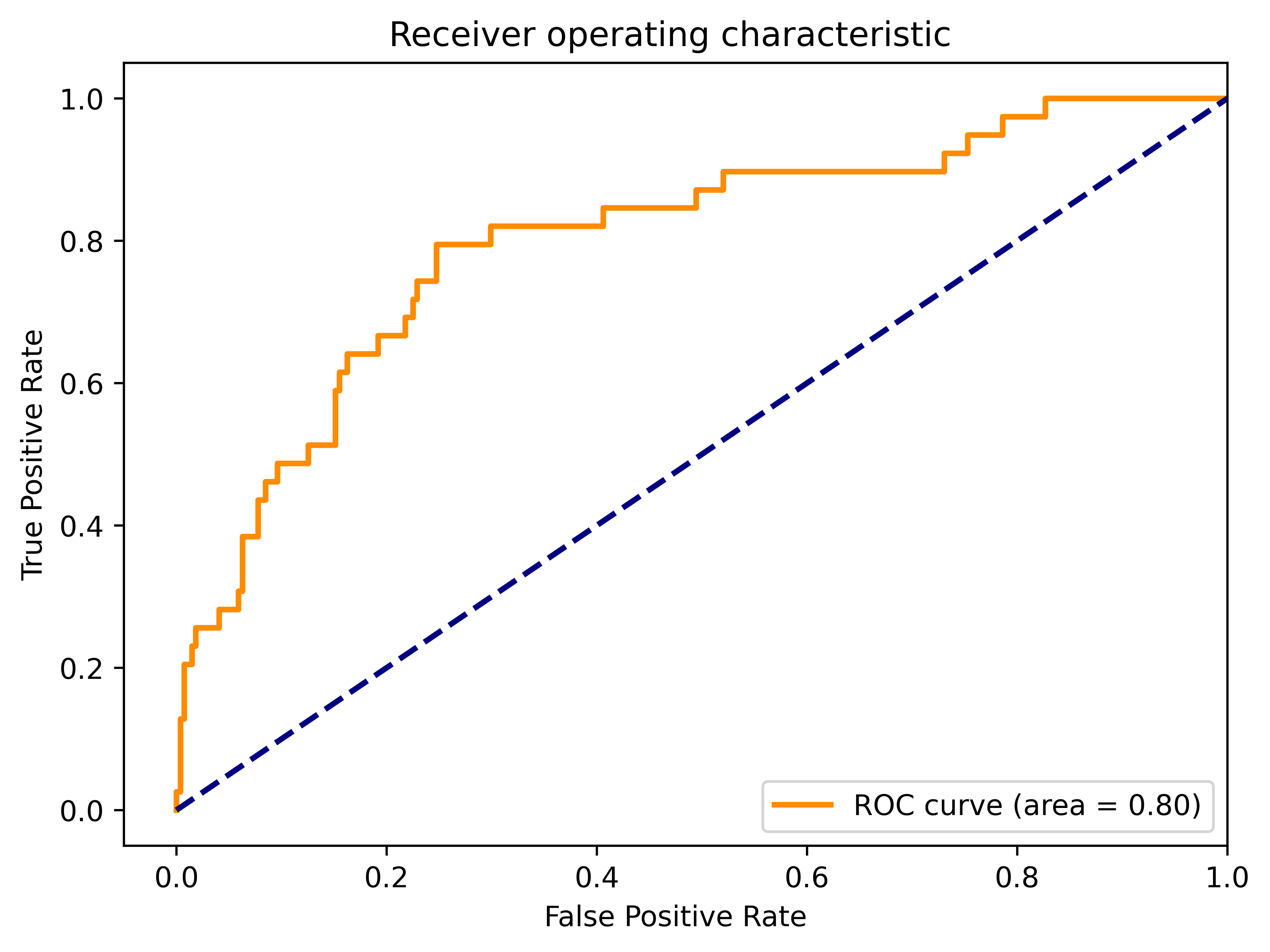


Supplementary figure 3 Receiver operating characteristic curves of XGBoost model with LASSO selection features (AUC = 0.80)


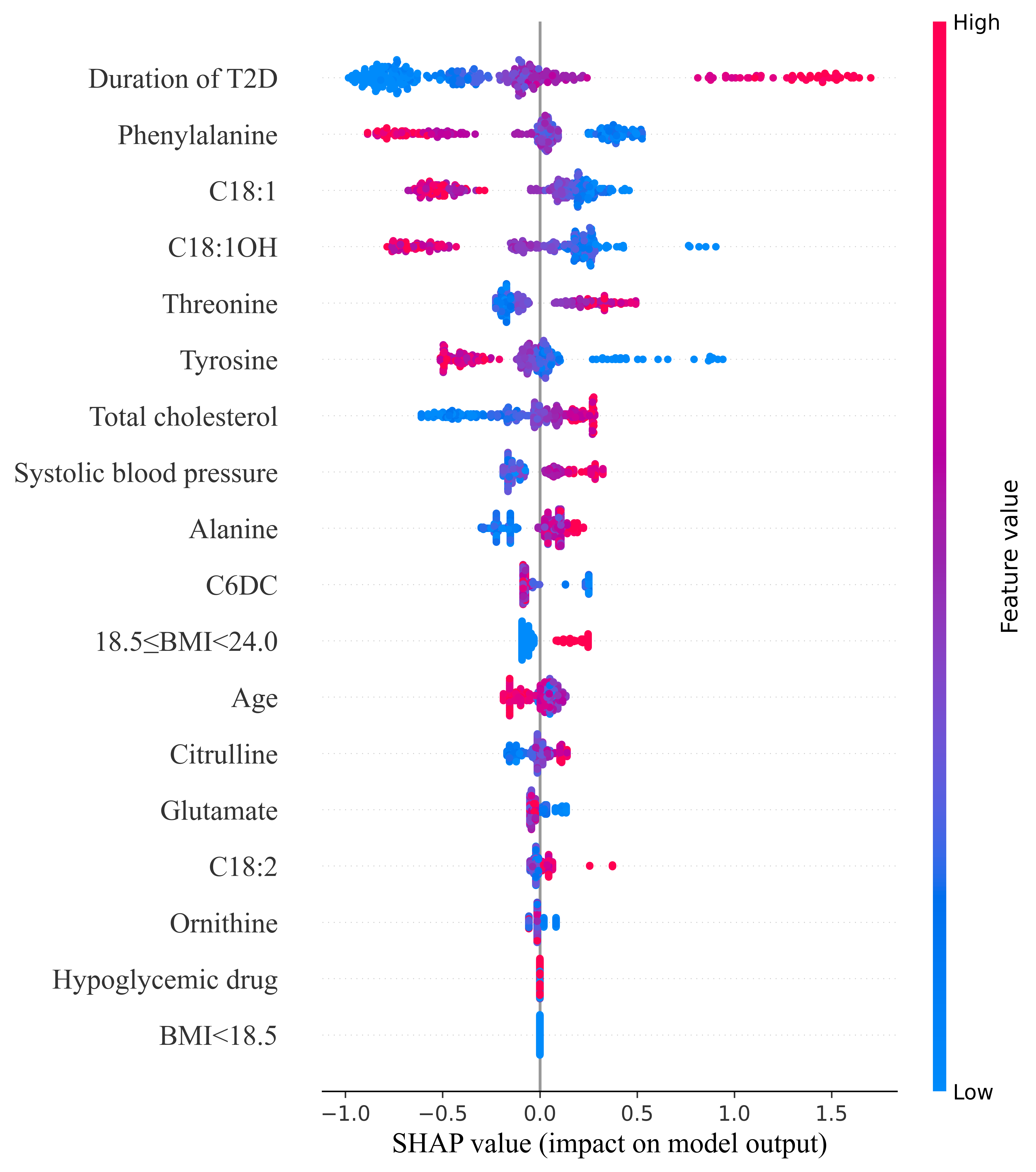


Supplementary figure 4 SHAP summary plot of XGBoost model with LASSO selection features after attribute scaling.
